# Ground glass nodules with scattered or eccentric island‐shaped consolidations may have poor outcomes

**DOI:** 10.1002/cai2.48

**Published:** 2023-02-23

**Authors:** Ming Li, Junjie Xi, Huan Zhang, Xing Jin, Zhuoyang Fan, Cheng Zhan, Mingxiang Feng, Lijie Tan, Qun Wang

**Affiliations:** ^1^ Department of Thoracic Surgery, Zhongshan Hospital Fudan University Shanghai China; ^2^ Cancer Center, Zhongshan Hospital Fudan University Shanghai China; ^3^ Department of Interventional Radiology, Zhongshan Hospital Fudan University Shanghai China

**Keywords:** lung adenocarcinoma, ground glass opacity, central island‐shaped, scattered or eccentric island‐shaped, prognosis

## Abstract

**Background:**

To explore the effect of scattered or eccentric shaped types of ground glass opacity (GGO) on outcomes of patients with solid‐dominant peripheral lung adenocarcinoma.

**Methods:**

We evaluated patients with solid‐dominant peripheral lung adenocarcinoma, who underwent radical surgery at Zhongshan Hospital, Fudan University, between January 2013 and December 2015. Morphologically heterogeneous solid‐dominant lung adenocarcinoma in imaging findings was based on the last preoperative computed tomography (CT) scans. Endpoints were recurrence‐free survival (RFS) and overall survival (OS). Kaplan–Meier analysis and the log‐rank test were used to estimate survival differences. Impact factors were assessed by univariable logistic regression analysis.

**Results:**

We retrospectively collected data from 200 patients, including 170 patients with central island‐shaped CT imaging, 18 patients with scattered shaped CT imaging, and 12 patients with eccentric shaped CT imaging. Eleven patients experienced recurrence or metastases. Kaplan–Meier survival curves showed significant survival differences between the central island‐shaped type and scattered shaped or eccentric shaped type for OS (c‐stage IA: 5‐year OS: 100% vs. 92.1%; HR = 0.019, *p* = 0.0025; p‐stage IA: 100% vs. 95.2%; HR = 0.146, *p* = 0.1139) and RFS (c‐stage IA: 5‐year RFS: 100% vs. 59.7%; HR = 0.001, *p* < 0.0001; p‐stage IA: 100% vs. 64.5%; HR < 0.001, *p* < 0.0001). Univariable logistic regression analysis showed that scattered and eccentric shaped types were related to poor outcomes (*p* < 0.012, odds ratio = 18.8).

**Conclusions:**

Relative spatial position of GGO and solid components may affect patient outcomes. Patients with scattered or eccentric shaped GGO may have a poor prognosis.

## Graphical abstract

In this single‐center retrospective study, patients with scattered‐ or eccentric‐shaped ground glass opacity (GGO) were more likely to have poor outcomes and up‐stage after surgery. Patients with central island‐shaped and scattered/eccentric GGO had different pathology subtypes distribution. Micropapillary and solid subtypes more commonly occurred in the scattered‐ or eccentric‐shaped GGO.

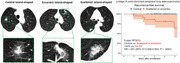

**Abbreviations**
c‐Stage: clinical stage
CI: confidence interval
CT: computed tomography
CTR: consolidation/tumor ratio
EGFR: epidermal growth factor receptor
GGO: ground glass opacity
HR: hazard ratio
HU: Hounsfield unit
ICC: intraclass correlation coefficient
JCOG: Japan Clinical Oncology Group
OS: overall survival
p‐Stage: pathological stage
RFS: recurrence‐free survival
WHO: World Health Organization

## 1 INTRODUCTION

The presence of GGO components is generally believed to be a protective factor [1]. GGO is a noninvasive component, and partly solid nodules with GGO have a significantly different prognosis from fully solid nodules [2]. Patients with GGO lesions have a significantly better prognosis after surgical resection than those with pure‐solid lung cancer. In accordance with the most recent results of Japan Clinical Oncology Group (JCOG) studies, patients with solid‐dominant peripheral lung adenocarcinoma (consolidation to tumor ratio [CTR] >0.5) also have a favorable prognosis [1, 3]. Notably, however, lung adenocarcinoma presenting as GGO may not always have a favorable prognosis. Patients with early‐stage lung adenocarcinoma who present as GGO may experience recurrence after lobectomy or partial resection. There are even some patients whose postoperative pathology has confirmed occult lymph node metastasis. Interestingly, beyond the pathological subtypes (micropapillary and solid), morphologically heterogeneous solid‐dominant lung adenocarcinoma in imaging findings may also be associated with a poor prognosis.

In the present study, we retrospectively studied morphologically heterogeneous solid‐dominant peripheral lung adenocarcinoma in 200 patients, including 11 who underwent recurrence and metastasis after surgery to indicate whether the relative position of solid and GGO components affects patient outcomes. The morphological appearance of solid‐dominant peripheral lung adenocarcinoma on preoperative computed tomography (CT) was postulated to be associated with the prognosis after classification into two types: central island‐shaped and scattered or eccentric island‐shaped.

## 2 METHODS

### 2.1 Patients and follow‐up

In total, 200 patients with solid‐dominant peripheral lung adenocarcinoma, who underwent radical surgery at Zhongshan Hospital, Fudan University, between January 2013 and December 2015, were analyzed retrospectively. Before surgery, all patients received a thin‐section CT scan (collimation ≤1.5 mm). Only primary lesions were analyzed. The selection criteria were as follows: [1] CTR > 0.5 and maximum tumor diameter ≤3 cm; [2] final pathological report confirmed the diagnosis of primary lung adenocarcinoma/invasive lung adenocarcinoma; [3] R0 resections were achieved. Exclusion criteria were as follows: [1] patients with missing radiological or clinicopathological data; [2] patients who received neoadjuvant therapy before surgery; [3] patients who had a history of tumors in other organs. Finally, 200 patients were included. All operations were carried out by thoracic surgeons in our hospital. Most patients underwent a standard lobectomy, whereas partial resection (segmentectomy or wedge resection) was also performed for some patients. A minimum of three N2 stations sampled or completed lymph node dissections were the routine schedule for all patients. Postoperative data were collected through outpatient follow‐up and annual telephone follow‐up. The last follow‐up date was November 2020. Patients were censored at the last follow‐up if the patient was still alive or lost to follow‐up. Patients were excluded if their recurrence, metastatic status, or follow‐up times were unknown.

### 2.2 CT measurement

Lung windows were set at a window width of 1000 Hounsfield units (HU) and a window level of −600 HU. GGO is defined as a hazy opacity in the lung without obscuring the underlying bronchial structures or pulmonary vessels. The maximum diameter of the solid portion and total tumor in the lung window were measured. CTR was defined as the ratio of the maximum size of consolidation to the maximum tumor size in the lung window. The intraclass correlation coefficient was used to assess consistency between two quantitative measurements. For multiple GGOs, dominant lesions were investigated. The findings of preoperative thin‐section CT scans were reviewed independently by two radiologists and two thoracic surgeons. Disagreements were resolved by another radiologist or thoracic surgeon. All patients had follow‐up examination by thin‐section CT scans at least three or more months apart (from the first diagnosis) before surgery in our hospital. Morphologically heterogeneous solid‐dominant lung adenocarcinoma of imaging findings in the lung window was based on the last preoperative CT scan. During the preoperative follow‐up, we compared the CT scan findings to clarify whether the radiographic morphology of patients changed between the central and scattered or eccentric island‐shaped types.

### 2.3 Statistical analyses

Statistical analyses were performed using SPSS Statistics 22.0 (IBM, Inc) and R version 3.3.2 (R Foundation for Statistical Computing). We used the *t*‐test and Wilcoxon test for continuous variables and Fisher's exact or *χ* ^2^ tests for categorical variables to test for differences. We used logistic regression to analyze the relationship between a binary outcome or categorical outcome and multiple influencing factors. Statistical significance was set at a two‐sided *p* < 0.05 Kaplan–Meier and log‐rank tests were used to analyze and compare overall survival (OS) and recurrence‐free survival (RFS). RFS was defined as the interval from the surgery date to the time of the first recurrence, metastasis, or last follow‐up.

## 3 DATA AVAILABILITY STATEMENT

The data underlying this article cannot be shared publicly because of ethical and privacy reasons. The data will be shared at reasonable request to the corresponding author.

## 4 RESULTS

### 4.1 Patients and follow‐up

**Figure 1:**
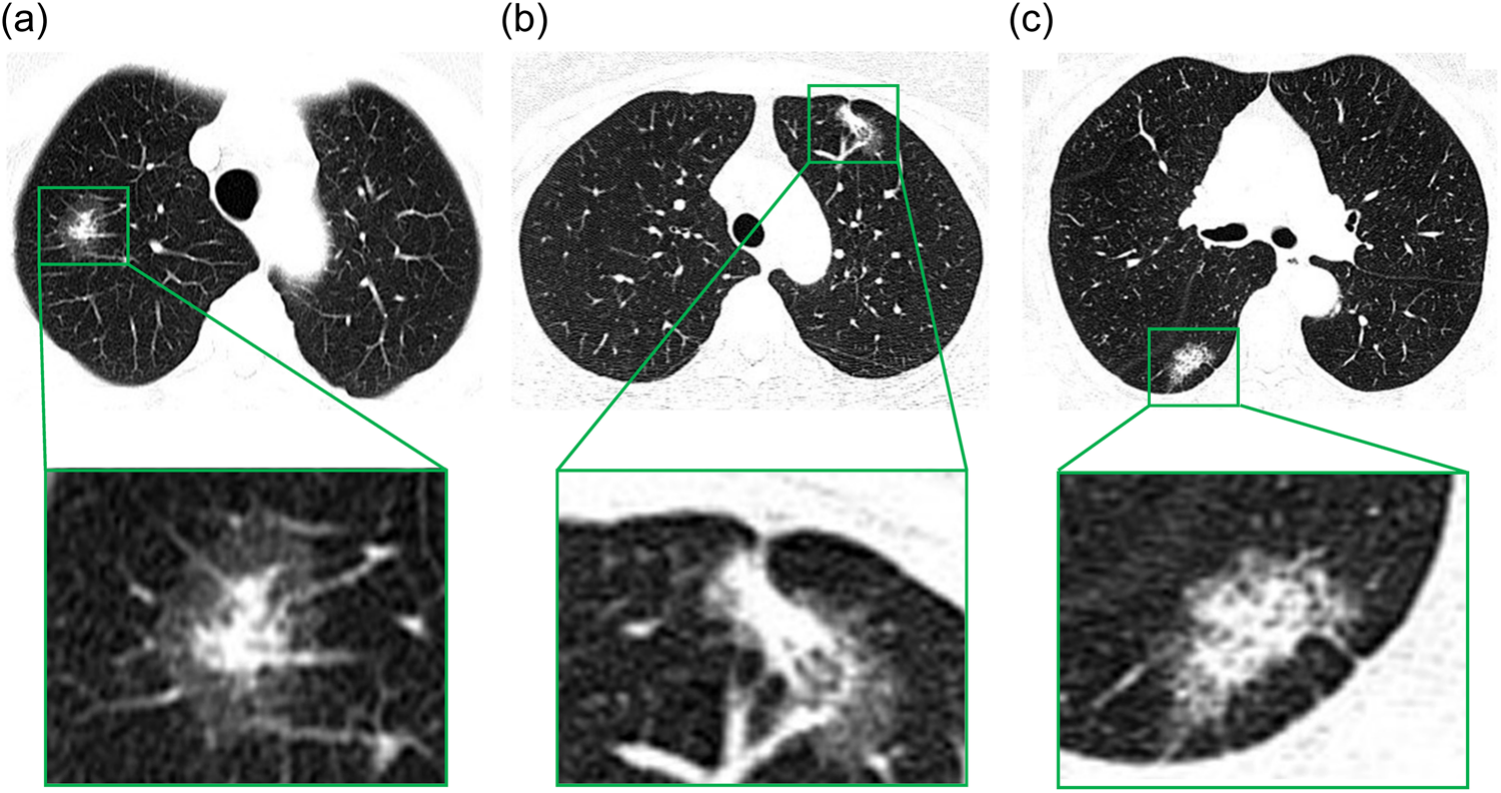
Thin‐section computed tomography images of the central island type, (a) eccentric island type, (b) and scattered shaped type. (c) The relative spatial location of ground glass opacity and the solid component can be seen. [lung window, window width: 1000 Hounsfield units (HU), window level: −600 HU].

We retrospectively collected data from 200 solid‐dominant (CTR > 0.5) peripheral lung adenocarcinoma patients who underwent radical surgery with 170 patients showing central island‐shaped CT imaging, 18 patients showing scattered shaped CT imaging, and another 12 patients showing eccentric shaped CT imaging. Representative examples of thin‐section CT images of central island‐shaped, scattered, and eccentric island‐shaped types are shown in Figure 1. The baseline characteristics of the two groups are shown in Table 1. No significant differences were found between groups in terms of age, sex, CTR, smoking history, surgical procedures, and lymph node dissection (Table 1). The intraclass correlation coefficient (C, 1) model showed a satisfactory correlation coefficient (0.972, 95% CI: 0.918–0.992) in CT measurements. No patients had pathological evidence of spread through air spaces. The median follow‐up time was 48.6 months, and the maximum follow‐up time was 108 months. Over the course of follow‐up, 11 patients experienced recurrence or metastases (2 local recurrence and 9 distant metastases) with 2 patients being scattered and 9 patients being the eccentric island‐shaped type, while no patient had the central island‐shaped type.

**Table 1:** Baseline information of 200 patients with solid‐dominant peripheral lung adenocarcinoma

	Central island‐shaped	Scattered or eccentric island‐shaped	*p‐*value
**Total evaluated**	170	30	
**Age (years)**			.851
Mean ± SD	61.4 ± 8.4	61.0 ± 10.7	
**Sex**			.113
Male	56 (32.9%)	6 (20.0%)	
Female	114 (67.1%)	24 (80.0%)	
**GGO size, mm**			.002
Mean ± SD	18.5 ± 6.5	22.8 ± 7.4	
**Solid size, mm**			<.001
Mean ± SD	11.9 ± 4.6	15.2 ± 6.0	
**CTR**			.273
Mean ± SD	0.64 ± 0.10	0.67 ± 0.10	
**Smoking history**			.240
No	154 (90.6%)	29 (96.7%)	
Yes	16 (9.4%)	1 (3.3%)	
**Pleural invasion**			<.001
No	165 (97.1%)	21 (70.0%)	
Yes	5 (2.9%)	9 (30.0%)	
**Procedure**			.769
Lobectomy	119 (70.0%)	20 (66.7%)	
Segmentectomy	15 (8.8%)	4 (13.3%)	
Wedge resection	36 (21.2%)	6 (20.0%)	
**Lymph‐nodes dissection**			.715
Mediastinal node dissection	51 (30.0%)	10 (33.3%)	
Systematic sampling	119 (70.0%)	20 (66.7%)	
**Clinical T stage**			.009
T1a	77 (45.3%)	7 (23.3%)	
T1b	83 (48.8%)	17 (56.7%)	
T1c	10 (5.9%)	6 (20.0%)	
**Pathologic T stage**			<.001
T1a	53 (31.2%)	5 (16.7%)	
T1b	90 (52.9%)	9 (30.0%)	
T1c	20 (11.8%)	7 (23.3%)	
T2a	7 (4.1%)	9 (30.0%)	
**Recurrence**			<.001
No	170 (100.0%)	19 (63.3%)	
Yes	0 (0.0%)	11 (36.7%)	
**Predominant subtype (IASLC/ATS/ERS Classification)**			<.001
Ais/MIA	25 (14.7%)	0 (0.0%)	
With lepidic component, but without micropapillary or solid components	31 (18.2%)	4 (13.3%)	
With acinar or papillary component, but without lepidic, micropapillary or solid components	112 (65.9%)	21 (70.0%)	
With micropapillary or solid components	0 (0.0%)	5 (16.7%)	
Invasive mucinous adenocarcinoma	2 (1.2%)	0 (0.0%)	

*Note*: *p* Value determined by the paired *t*‐test or Wilcoxon signed‐rank test for continuous variables and Fisher's exact or *χ* ^2^ test for categorical variables to test for differences.

Abbreviations: Ais, adenocarcinoma in situ; CTR, consolidation tumor ratio; GGO, ground glass opacity; IASLC/ATS/ERS, International Association for the Study of Lung Cancer, American Thoracic Society, and European Respiratory Society; MIA, minimally invasive adenocarcinoma.

**Figure 2:**
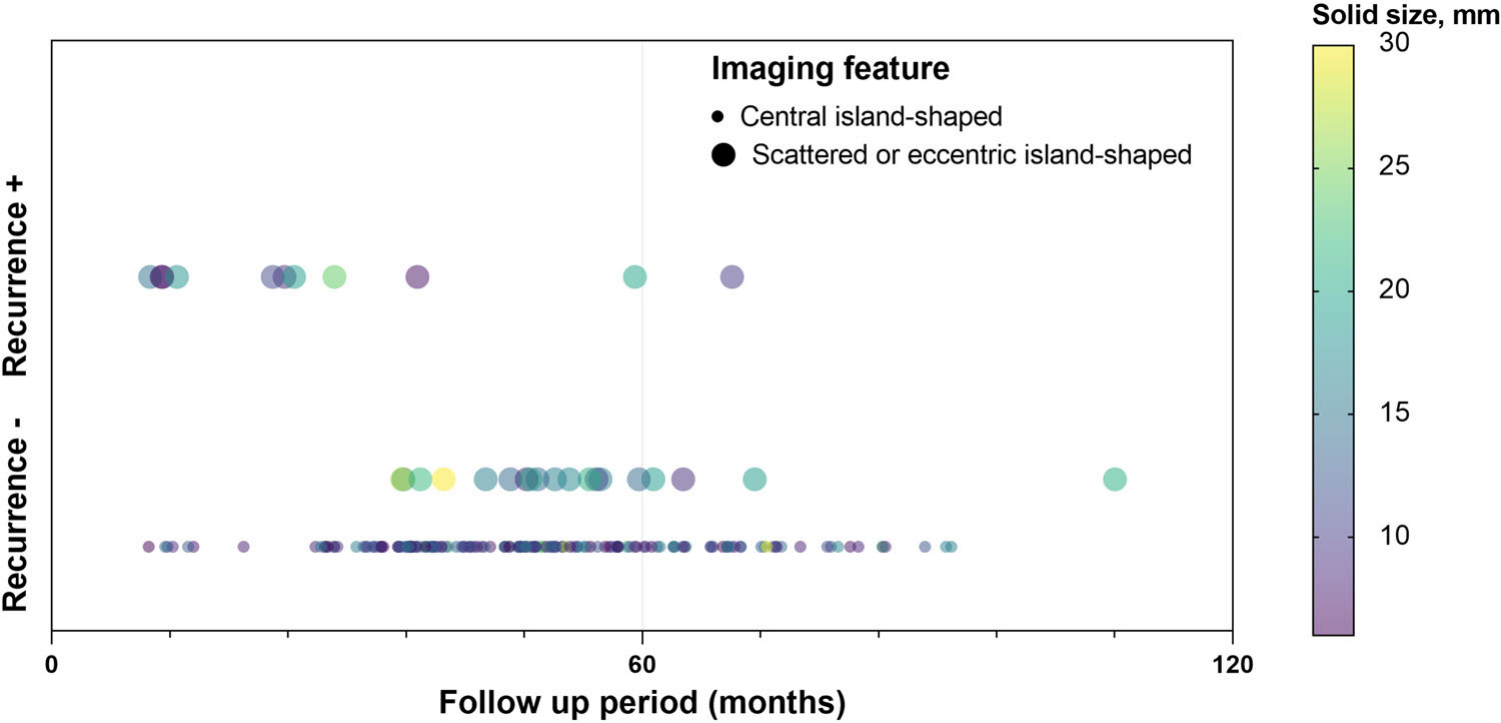
Specific timeline of follow‐up after grouping by recurrent status and imaging features. Each round dot indicates a patient. Colors indicate different solid sizes, and dot sizes represent the two types of radiographic morphology: central and scattered or eccentric island‐shaped types.

Figure 2 depicts the specific timeline of follow‐up after grouping by whether recurrent metastasis had occurred. Each round dot represents a patient. Colors indicate different solid sizes, and dot sizes represent the two types of radiographic morphology: central island‐shaped and scattered or eccentric island‐shaped types. During the study period, 11 of 200 patients (5.5%) (all with scattered or eccentric island‐shaped types) had developed recurrence or metastasis. No patients experienced recurrence or metastasis in the central island‐shaped group. Intergroup comparison revealed a statistically significant difference in RFS (*p* < 0.001, *χ* ^2^ = 69.17). In scattered and eccentric shaped groups, the median RFS was 69.1 months (95% CI: 53.3–85.0 months). Median RFS for central island‐shaped patients had not been attained after a median follow‐up of 47.8 months, indicating that patients with scattered or eccentric shaped GGO may have a worse prognosis than those with the central island‐shaped type. Furthermore, there was no discernible difference in prognosis between scattered and eccentric shaped groups.

### 4.2 Prognostic value of scattered and eccentric shaped types

**Figure 3:**
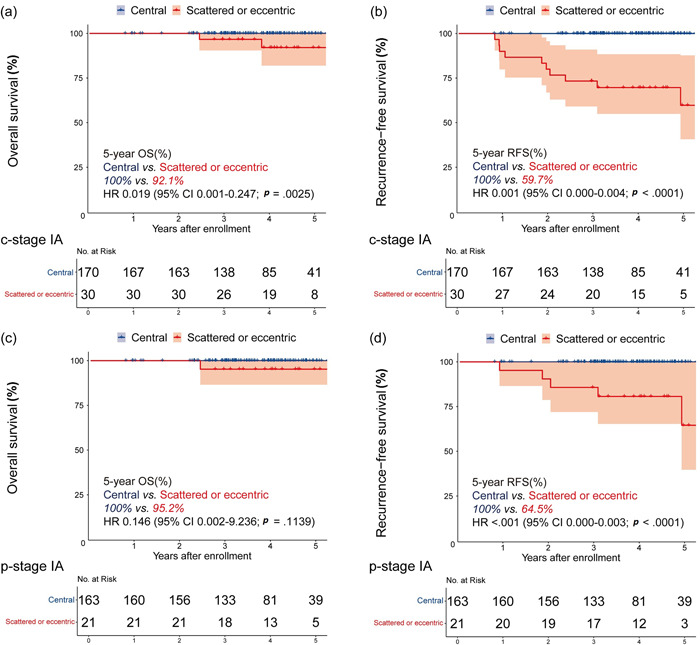
Survival outcomes of central island‐ and scattered or eccentric shaped types in c‐stage IA solid‐dominant peripheral lung adenocarcinoma for OS, ([a] 5‐year OS: 100% vs. 92.1%; HR = 0.019, 95% CI: 0.001–0.247, *p* = 0.0025) and RFS, ([b] 5‐year RFS: 100% vs. 59.7%; HR = 0.001, 95% CI: 0.000–0.004, *p* < 0.0001). For patients in p‐stage IA, OS ([c] 5‐year OS: 100% vs. 95.2%; HR = 0.146, 95% CI: 0.002–9.236, *p* = 0.1139) and RFS ([d] 5‐year RFS: 100% vs. 64.5%; HR < 0.001, 95% CI: 0.000–0.003, *p* < 0.0001). HR, hazard ratio; OS, overall survival; RFS, recurrence‐free survival.

To investigate whether scattered and eccentric shaped types were associated with prognosis, we analyzed the RFS and OS of patients with solid‐dominant peripheral lung adenocarcinoma. The primary endpoint was RFS, and the secondary endpoint was OS. OS may be influenced by postoperative adjuvant therapy when patients have developed recurrence or metastasis. Survival curves are presented in Figure 3. We conducted survival analyses separately by the clinical stage (c‐stage) and pathological stage (p‐stage). For patients in c‐stage IA, Kaplan–Meier survival curves showed significant survival differences between central island‐ and scattered or eccentric shaped types for OS (5‐year OS: 100% vs. 92.1%; HR = 0.019, 95% CI: 0.001–0.247, *p* = 0.0025) and RFS (5‐year RFS: 100% vs. 59.7%; HR = 0.001, 95% CI: 0.000–0.004, *p* < 0.0001) (Figure 3a,b). For patients in p‐stage IA, survival analysis also showed that the scattered or eccentric shaped type of GGO significantly indicated worse RFS (5‐year RFS: 100% vs. 64.5%; HR < 0.001, 95% CI: 0.000–0.003, *p* < 0.0001). However, no significant difference in OS (5‐year OS: 100% vs. 95.2%; HR = 0.146, 95% CI: 0.002–9.236, *p* = 0.1139) was found between the two types.

We preliminary performed binomial logistic regression to determine the effect of scattered and eccentric shaped types on emergence of recurrence or metastasis. Univariable logistic regression analysis showed that scattered and eccentric shaped types were related to poor outcomes (*p* < 0.012, odds ratio = 18.8, 95% CI: 1.884–187.2). However, a Cox regression model based on adjusted events could not be well developed for this outcome because of the lack of events.

### 4.3 Distribution of pathological subtypes

**Figure 4:**
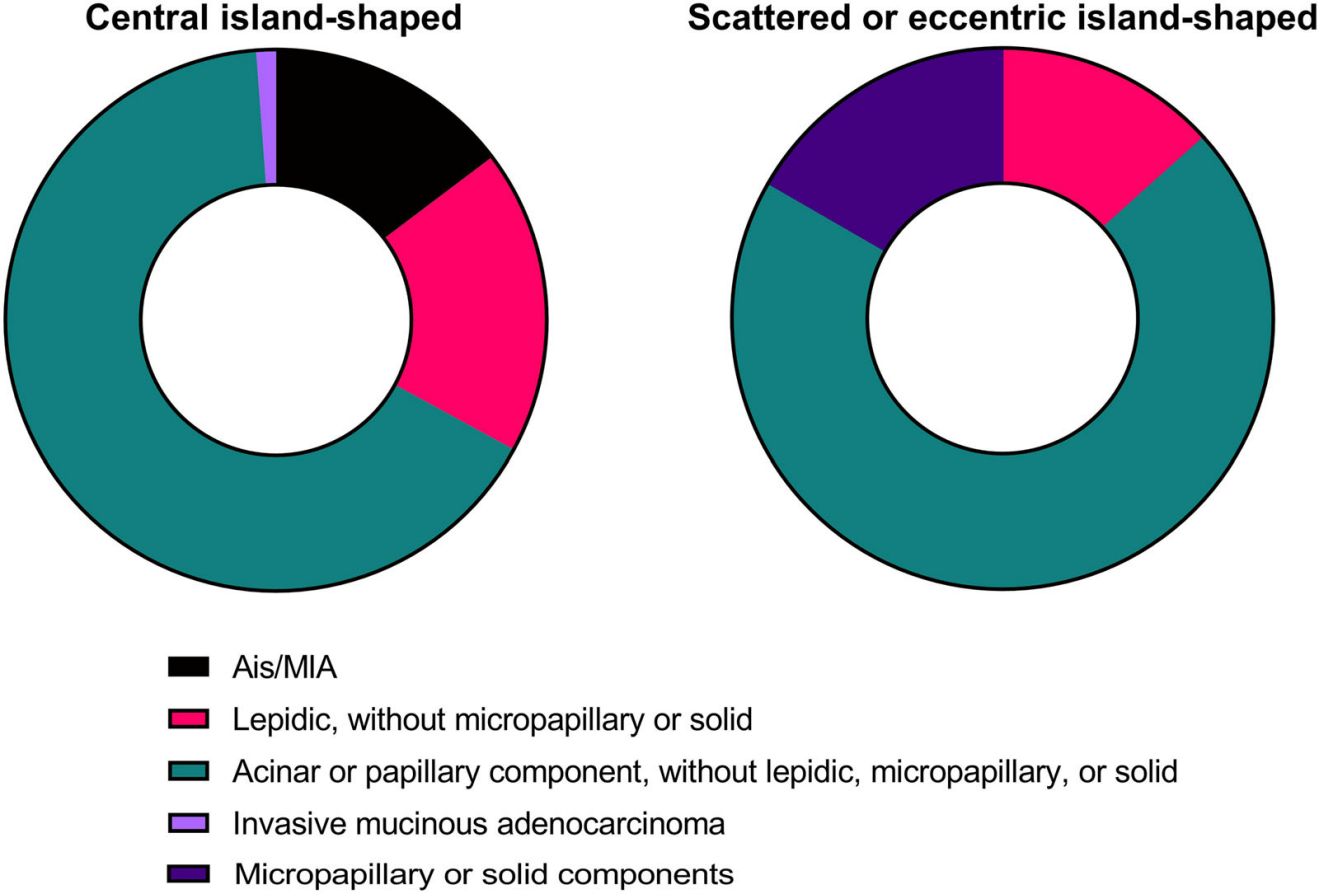
Detailed distribution of pathological subtypes of patients with central island‐shaped and scattered or eccentric island‐shaped types. Ais, adenocarcinoma in situ; MIA, minimally invasive adenocarcinoma.

A difference in the distribution of pathological subtypes was also found between central island‐ and scattered or eccentric shaped types (*p* < 0.001) (Figure 4). Adenocarcinoma in situ or minimally invasive adenocarcinoma was seen only among patients with the central island‐shaped type and never among patients with the scattered or eccentric shaped type. Conversely, adenocarcinoma with micropapillary or solid components was seen only among patients with the scattered or eccentric shaped type. Moreover, a lepidic pattern without a micropapillary or solid component was more common in patients with the central island‐shaped type. No difference in subtype distribution of patients with acinar or papillary components without lepidic, micropapillary, or solid components was observed between the two types. Logistic regression analysis indicated that the scattered or eccentric shaped morphology was related to solid/micropapillary patterns (*p* = 0.015, odds ratio = 4.661, 95% CI: 1.357–16.013).

### 4.4 Details of patients with recurrence or metastasis

Table 2 shows the details of the 11 patients with recurrent metastases. Their ages ranged from 42 to 72 years with a median of 63 years. Three men (4.8% of all male individuals) and eight women (5.8% of all female individuals) were among the patients. Only one of the patients had ever smoked. The median CTR of these patients with metastatic recurrence was 0.68 (range, 0.60–0.83). Preoperative imaging revealed no signs of lymph node metastasis. Surprisingly, the postoperative pathological T stage of five patients was more advanced than the clinical T stage, and two patients had N2 nodal metastases (Cases 1 and 10).

**Table 2:** Details of the 11 patients with recurrence or metastases, including clinicopathological characteristics, treatment after relapse, and imaging features

	Case 1	Case 2	Case 3	Case 4	Case 5	Case 6	Case 7	Case 8	Case 9	Case 10	Case 11
Age, years	58	67	42	60	58	76	63	63	55	72	67
Sex	Male	Female	Female	Female	Female	Male	Female	Female	Female	Female	Male
Smoking	No	No	No	No	No	No	Yes	No	No	No	No
cT‐stage	T1a	T1b	T1a	T1a	T1a	T1b	T1b	T1c	T1a	T1b	T1b
cN‐stage	N0	N0	N0	N0	N0	N0	N0	N0	N0	N0	N0
GGO‐size, mm	12	30	11	8.5	15	17	22	32	13	25	24
Solid‐size, mm	10	20	7	7	9	11	15	24	7	19	18
CTR	0.83	0.67	0.64	0.82	0.6	0.65	0.68	0.75	0.54	0.76	0.75
Location	RML	LUL	LUL	RUL	LLL	LUL	RUL	LUL	RUL	RUL	LLL
Procedure	Lobe	Lobe	Segment	Lobe	Wedge	Segment	Lobe	Lobe	Lobe	Lobe	Segment
pT‐stage	pT2a	pT1c	pT1a	pT2a	pT2a	pT1b	pT2a	pT2a	pT1a	pT1b	pT2a
pN‐stage	N2	N0	N0	N0	N0	N0	N0	N0	N0	N2	N0
Pleural invasion	PL1/2	PL1/2	PL0	PL1/2	PL1/2	PL0	PL1/2	PL0	PL0	PL0	PL1/2
WHO classification	Invasive	Invasive	Invasive	Invasive	Invasive	Invasive	Invasive	Invasive	Invasive	Invasive	Invasive
Predominant subtype	Acinar/solid	Acinar	Lepidic	Papillary/solid	Lepidic/acinar	Acinar/papillary	Acinar	Acinar	Lepidic	Acinar	Acinar
EGFR‐mutant	L858R	19del	L858R	L858R	NA	NA	NA	L858R	NA	L858R	19del
RFS, month	69	59	37	11	24	22	10	29	11	25	13
Recurrence site	Lung	Bone/lung/liver	Reluctant to disclose	liver	Reluctant to disclose	Reluctant to disclose	Lung/bone	bone	Lung	Bone	Lung
Adjuvant treatment after relapse	Gefitinib + wedge resection	Gefitinib/osimertinib	Reluctant to disclose	Reluctant to disclose	Reluctant to disclose	Reluctant to disclose	Pem/Cis	Osimertinib	Wedge resection + SBRT	Gefitinib	Osimertinib
Imaging features	Eccentric island‐shaped	Scattered	Scattered	Eccentric island‐shaped	Scattered	Scattered	Scattered	Scattered	Scattered	Scattered	Scattered

Abbreviations: EGFR, epidermal growth factor receptor; RFS, recurrence‐free survival; SBRT, stereotactic body radiotherapy; WHO, World Health Organization.

Owing to pleural infiltration confirmed by postoperative pathology, T‐staging was upgraded in all pathological T2a patients. Moreover, two patients with postoperative lymph node metastases had occult lymph node metastases. Pathological subtypes of most tumors were acinar and lepidic. However solid or papillary components were detected in three of the 11 patients who developed recurrent metastases (Cases 1, 4, and 6). Seven patients had an epidermal growth factor receptor (EGFR) mutation (5 patients had an EGFR exon 21 L858R mutation, and two patients had a deletion 19 mutation). The median duration for metastasis or recurrence in these patients was 24 months (range, 10–69 months). Three patients (27.3%) had lung recurrences, and eight patients (72.7%) acquired distant metastasis, including bone metastases (*n* = 2), liver metastases (*n* = 1), multiple metastases (*n* = 2), and other nonspecific sites (*n* = 3).

Case 1 was treated with gefitinib and secondary segment resection, Case 11 was treated with osimertinib, and Case 9 was treated with SBRT for intrapulmonary recurrence. Osimertinib and gefitinib were used to treat one patient with numerous metastases, whereas Pem and Cis were used to treat the other (Case 7). Two patients with bone metastases were treated with EGFR TKIs with case 8 receiving osimertinib and Case 10 receiving gefitinib.

In this retrospective observational study of patients with solid‐dominant peripheral lung adenocarcinoma (CTR > 0.5) who had undergone radical surgery, we found that the morphological characteristics of the nodes in the preoperative CT findings may be related to the risk of recurrent metastasis. Specifically, patients with scattered or eccentric shaped GGO on CT may have a higher risk of postoperative recurrent metastases than those with central island‐shaped nodules. At the cutoff date for follow‐up, three of the 11 patients died because of postsurgical relapse, metastases, or other diseases.

## 5 DISCUSSION

Recently, we focused on the results of JCOG0802/WJOG4607L1, which showed that segmentectomy could be the standard surgical procedure for patients with small peripheral nonsmall cell lung cancer [4]. However, maybe not all ground glass nodules are good tumors with good outcomes. In this study, we found that patients with scattered shaped or eccentric shaped GGO may have poor outcomes and were more likely to undergo progression after surgery.

Many studies have confirmed that GGO is a heterogeneous group of tumors with different biological behaviors and prognoses. For example, Lu and colleagues demonstrated that GGO is significantly different from solid tumors in terms of proliferation, angiogenesis, and the immune microenvironment by single cell sequencing [5]. Although the prognosis of GGO is generally better than that of solid adenocarcinoma, complete avoidance of postoperative recurrent metastases also appears impossible for all GGO patients. Investigating the risk factors that postoperative recurrent metastasis in GGO patients is meaningful and necessary.

Mounting evidence shows that CT imaging has reference value in determining the benignity or malignancy of GGO [6, 7, 8, 9]. Some CT imaging markers, such as the nodule size, vascular convergence sign, and pleural indentation, predict the biological activity and prognosis of GGO to a degree. For example, CTR is a well‐known risk factor that affects GGO recurrence and the patient survival after surgery. Large CTR nodes are generally believed to predict a poor prognosis [10]. Several studies have also found that the nodule shape also influences GGO [11, 12]. The current study focused only on the general morphology of nodules, such as whether they are regular in shape and have lobulation or spiculation signs. However, no study has examined the prognostic value of the relative position of solid and GGO components. We have previously reported that a GGO component influences a favorable prognosis [2]. More importantly, our daily practice has demonstrated that, although the solid component plays a role in malignant behavior, the GGO component may be a decisive and protective factor. Thus, the relative spatial position may affect this protective effect and change the distribution of invasive components.

Our study revealed that morphological features may be another predictor of prognosis for GGO patients. We classified 200 solid‐dominant peripheral lung adenocarcinoma patients into 3 morphologies by the relationship between the location of solid and ground glass components on thin‐section CT, including scattered, eccentric, and central island‐shaped types. Of the 200 patients, 11 experienced recurrences during the follow‐up period. Thus, RFS was used as the endpoint of this study. We found that the 11 patients who experienced recurrent metastasis were all scattered or eccentric shaped types. Interestingly, both had a characteristic that the ground glass component did not wholly encase the solid component (i.e., irregular form of the solid component). We confirmed a statistically significant difference in prognosis between the two groups of GGO grouped by morphology by survival analysis, indicating that patients with scattered or eccentric shaped types may have a higher risk of recurrence and metastasis. However, we do not know why this morphological difference occurs and the reason related to outcomes. It is possible that this difference may also be associated with the different distributions of pathological subtypes between the two types. In accordance with the grading system for invasive pulmonary adenocarcinoma from the International Association for the Study of Lung Cancer Pathology Committee, tumors that are solid or micropapillary predominant are classified as high grade with a poor outcome [13]. Several studies have indicated that patients with solid and/or micropapillary patterns have a poor prognosis, even if their patterns are not predominant. Interestingly, the scattered or eccentric shaped morphology was related to solid/micropapillary patterns that indicate a poor outcome [14]. However, further studies are needed to explore the mechanism underlying the correlation.

The present study had several limitations. This was a relatively small, single‐center retrospective study. Thus, more verification with larger sample sizes and multicenter studies should be conducted. Moreover, because of the small sample size and events, we did not analyze the difference in prognosis between scattered and eccentric shaped types, which may also be of interest. Furthermore, in this analysis, we focused only on RFS of patients. Analyzing the effect of morphological characteristics of GGO on OS was challenging because the follow‐up time was not sufficiently long, which may not accurately reflect patient survival. We analyzed the effect of CT morphological features of GGO on recurrence of metastasis in postoperative patients. We propose a possible novel predictor for the prognostic assessment of GGO patients. Our results should be verified in more extensive studies to improve the conclusions.

## 6 CONCLUSIONS

The relative spatial position of GGO and solid components may affect the prognosis of patients. Once GGO cannot wholly wrap the solid components or disperse the solid components of central concentration, it may be related to the micropapillary and solid pattern with a poor prognosis.

## AUTHOR CONTRIBUTIONS

**Ming Li**: Conceptualization (equal); data curation (equal); formal analysis (lead); methodology (equal); resources (equal); software (lead); visualization (lead); writing – original draft (lead). **Junjie Xi**: Conceptualization (equal); formal analysis (equal); investigation (equal); methodology (equal); project administration (equal); resources (equal); supervision (equal); writing – review & editing (equal). **Huan Zhang**: Conceptualization (supporting); data curation (equal); formal analysis (equal); methodology (equal); software (equal); visualization (equal); writing – original draft (equal). **Xing Jin**: Data curation (equal); formal analysis (supporting); methodology (supporting); resources (supporting); software (supporting); writing – original draft (supporting). **Zhuoyang Fan**: Data curation (equal); formal analysis (supporting); investigation (equal); methodology (supporting); visualization (supporting). **Cheng Zhan**: Funding acquisition (equal); investigation (equal); project administration (supporting); validation (equal); writing – review & editing (supporting). **Mingxiang Feng**: Conceptualization (supporting); funding acquisition (supporting); methodology (supporting); project administration (supporting); resources (supporting); writing – review & editing (supporting). **Lijie Tan**: Conceptualization (supporting); funding acquisition (equal); project administration (lead); resources (equal). **Qun Wang**: Conceptualization (lead); formal analysis (supporting); methodology (supporting); project administration (lead); resources (lead); writing – review & editing (supporting).

## CONFLICT OF INTEREST

The authors declare no conflict of interest.

## ETHICS STATEMENT

This study was approved by the institutional review board of Zhongshan Hospital, Fudan University (B2018‐137R, July 27, 2018).

## INFORMED CONSENT

Not applicable.

## Data Availability

The data that support the findings of this study are available on request from the corresponding author. The data are not publicly available because of privacy or ethical restrictions
